# Effects of *Lespedeza Cuneata aqueous extract* on testosterone-induced prostatic hyperplasia

**DOI:** 10.1080/13880209.2018.1564929

**Published:** 2019-02-06

**Authors:** Bong Kyun Park, Chang Won Kim, Jeong Eun Kwon, Manorma Negi, Yong Tae Koo, Sang Hun Lee, Dong Hyun Baek, Yoo Hun Noh, Se Chan Kang

**Affiliations:** a Clinical Medicine Division, Korea Institute of Oriental Medicine, Daejeon, Republic of Korea;; b Department of Oriental Medicine Biotechnology, Kyung Hee University, Yongin, Republic of Korea;; c Kwang-Dong Pharmaceutical Co., Ltd, Seoul, Republic of Korea;; d Famenity Co., Ltd, Gwacheon, Republic of Korea

**Keywords:** PCNA, inflammation, androgen receptor

## Abstract

**Context:***Lespedeza cuneata* G. Don (Fabaceae), has been used as a traditional treatment of various diseases. There is a report *L. cuneata* effects on hormone replacement therapy for endocrine-related disease. However, studies related to benign prostatic hyperplasia (BPH) have not been investigated.

**Objective:** The effects of *L. cuneata* aqueous extract (LCW) on testosterone-induced prostatic hyperplasia (TPH) were examined.

**Materials and methods:** Male Wistar rats (10 weeks, 330–350 g) were randomly divided to 6 groups (*n* = 6): Control group; TPH group (3 mg/kg, s.c, daily); TPH + LCW (25, 50, 100 mg/kg); TPH + Finasteride 10 mg/kg for 6 weeks. At the end of treatment, histological change of prostate, serum dihydrotestosterone (DHT) level, mRNA expression of 5α-reductase, inflammatory factors, proliferating cell nuclear antigen (PCNA) and fibroblast growth factor-2 (FGF-2) in prostate were examined. Then, LCW was treated with BPH-1, a human BPH cell line, at 25, 50, 100 μg/mL for 24 h and examine mRNA level of androgen receptor (AR) and prostate-specific antigen (PSA). In addition, the content of vicenin-2 was analyzed.

**Results:** LCW treatment of TPH inhibited serum DHT levels by 54.5, 51.2 and 54.1% and mRNA expression of 5α-reductase were inhibited 54.3, 61.3 and 73.6%, respectively. In addition, mRNA expression of inflammatory factors, PCNA and FGF-2 were decreased in the prostate of rats. Also, LCW attenuated mRNA level of AR and PSA in BPH-1 cell. The content of vicenin-2 in the LCW was analyzed to 0.89 mg/g.

**Discussion and conclusions:** Based on the results, LCW is a potential pharmacological candidate for the treatment of prostatic hyperplasia.

## Introduction

Benign prostatic hyperplasia (BPH) is a disease common in aging males. It is characterized by histological changes associated with significant growth of prostate stromal and epithelial cells leading to lower urinary tract symptoms (LUTS) (Gacci et al. 2012). Typical LUTS are increased urinary frequency, urgency incontinence and nocturia, and these symptoms are associated with increased risk of obstruction of the urethra, urinary retention and urinary infection (Miller & Tarter 2009). Medications currently used to treat BPH are 5α-reductase inhibitors and α_1_-adrenergic receptor antagonists (McVary 2007). The 5α-reductase inhibitors finasteride and dutasteride attenuate the development of BPH by inhibiting the production of dihydrotestosterone (DHT) from testosterone (Gravas & Oelke 2010). 5α-Reductase converts circulating testosterone in the prostate into the more potent androgen DHT, which is a metabolite of testosterone and an important mediator of prostate proliferation (Andriole et al. 2004). Drugs that target the α_1_-adrenergic receptor, such as doxazosin, terazosin and tamsulosin, are also used to treat BPH, and they relieve LUTS by relaxation of the neck of the bladder and smooth muscle in the prostate (Gilbert Jr et al. 2006; Shin et al. 2012). Both of these antagonists are effective in treating men with BPH. However, their use is considerably restricted due to their adverse effects, which include erectile dysfunction, loss of libido, dizziness and upper respiratory tract infection (Bullock & Andriole Jr 2006; Paba et al. 2011).

*Lespedeza cuneata* G. Don (Fabaceae) is a species of flowering plant native to Asia and eastern Australia. *L. cuneata* is used as a traditional herbal medicine for asthma, abscesses, breast cancer, and protection of liver and kidney function (Ahn 1998). Several studies have indicated that it has therapeutic effects on diabetes, low stamina and amblyopia (Huang 1998). Its bioactive components include β-sitosterol, quercetin, kaempferol, pinitol, avicularin, juglanin and trifolin, among others (Matsuura et al. 1978). These are known to have antioxidant, anti-inflammation and anticancer effects. In particular, β-sitosterol has the potential to inhibit BPH and high blood cholesterol levels (Wilt et al. 1999; Rudkowska et al. 2008). Roots and leaves of *L. cuneata* also contain minerals, amino acids, vitamins and flavonoids, suggesting that extracts of the leaves may have antioxidative and anti-inflammatory effects (Ding et al. 2006; Deng et al. 2007; Kim & Kim 2010). Although many previous studies have examined the pharmacological effects of *L. cuneata*, the pharmacological effects of an aqueous extract of *L. cuneata* (LCW) on testosterone-induced prostatic hyperplasia (TPH) have not been explored.

In this study, we, therefore, examined the inhibitory effects of an aqueous extract of *L. cuneata* (LCW) on the TPH rat model by measuring changes in prostate weight and the expression of DHT, 5α-reductase, and inflammatory cytokines as well as prostate histomorphology. Our results indicate that LCW may be a novel candidate medication to preventive prostatic hyperplasia.

## Materials and methods

### Chemicals and reagents

Standardized *L. cuneata* was obtained from Kwangdong Pharmaceutical Co., Ltd in January 2017. Specimens are stored in Kwangdong Pharmaceutical Co., Ltd (KP201701). The stem and leaves of *L. cuneata* were extracted in boiling water for 6 h. Filtered extracts were concentrated and powdered under reduced pressure. The yield was about 10.57%. The powder (LCW) was lyophilized and stored at 4 °C. Prior to this study, the genotoxicity of LCW was evaluated. Genotoxicity was assessed by the GLP organization (Korea Conformity Laboratories, Seoul, Korea), and no abnormalities were observed in the three tests: one for reversion mutations (GT17-00036), one for genetic mutations (GT17-00037) and a micronucleus test (GT17-00038), indicating no genotoxicity. Rat DHT enzyme-linked immunoassay (ELISA) MAX™ standards were obtained from BioLegend, Inc. (San Diego, CA). Unless otherwise indicated, all chemicals used in this research were purchased from Sigma-Aldrich Co. (St. Louis, MO).

### Animals and treatment

Experimental animals were 10-week-old male Wistar rats weighing 330–350 g obtained from Korea Laboratory Animal Co. (Daejeon, Korea). Rats were housed for 7 days prior to the experiment for acclimatization in solid-bottomed plastic cages designed to allow easy access to standard laboratory food and water. Mice were kept in sanitary ventilated animal rooms with a controlled temperature (25 ± 1 °C) and regular light cycle (12 h light/dark). Animal experiments were conducted in accordance with the current ethical regulations for animal care and use at Kyung Hee University (KHUASP(SE)-16-014). To prevent the influence of intrinsic testosterone, rats in all groups except the control group underwent bilateral orchiectomies 3 days prior to the administration of testosterone propionate. For the orchiectomies, animals were anesthetized by intraperitoneal injections of ketamine (0.05 mL/kg) and xylazine (0.05 mL/kg). The testis was exposed by performing a transverse resection on both scrota in the supine position, and the spermatic cord and blood vessels were ligated with 3-0 sutures and resected. To induce TPH, animals were randomly divided into a control group (*n* = 6) and TPH group (*n* = 6). Prostatic hyperplasia was induced in the latter group by daily subcutaneous injections of testosterone propionate (3 mg/kg, Sam Il Pharmaceuticals, Seoul, Korea) for 6 weeks. The effects of LCW were examined in a total of 12 rats for 6 weeks. Following the induction of prostatic hyperplasia, animals were randomly divided into four groups of four. Three of the groups were given an oral dose (25, 50 and 100 mg/kg, respectively) of LCW diluted in distilled water for 6 weeks, while the fourth group (control group) received finasteride 10 mg/kg for 6 weeks.

### Body and prostate weight

Body weight was measured weekly. After the last treatment, all animals were fasted overnight and sacrificed using CO_2_. Prostates were removed immediately and weighed. Relative prostate weight was calculated as the ratio of prostate weight to body weight. Percentage inhibition of the increase in prostate weight induced by LCW was determined as described in a previous study (Babu et al. 2010; Shin et al. 2012). The ventral lobe of the prostate was divided in half. One half was fixed using 10% neutral-buffered formalin and embedded in paraffin for histomorphology, while the other half was stored at −80 °C for other analyses.

### Prostatic index (PI)

The PI was calculated as the ratio of prostate weight to body weight.

### Percentage inhibition of prostate weight and prostatic index

Inhibition of the testosterone-induced increase in prostate weight and prostatic index were calculated as follows: 100 − [(T − NC)/(PC − NC) × 100], where NC, PC and T are negative control, positive control and treatment group values, respectively (Babu et al. 2010).

### Histopathological examination

To investigate morphological changes in the prostate, tissues were embedded in paraffin, cut into sections of 4 μm thickness, and stained with hematoxylin and eosin (H&E) solution (hematoxylin, Sigma MHS-16 and eosin, Sigma HT110-1-32). Tissues were subsequently mounted and cover slipped using mounting medium (Invitrogen, Carlsbad, CA) and then examined microscopically (Olympus, Tokyo, Japan).

### Serum analysis

At sacrifice, samples of whole blood were collected by cardiac puncture, and blood was allowed to clot for 30 min. Serum was then separated via centrifugation at 1500 *g* for 10 min. DHT, a marker of BPH present in the serum, was measured using ELISA kits for DHT (BioLegend, Inc.). All ELISA procedures were performed according to the manufacturer’s protocols.

### Isolation of total RNA and quantitative real-time PCR

Following the manufacturer’s protocol, total RNA was extracted from prostate tissue isolated from testosterone-induced rats orally administered LCW using Trizol reagent. Isolated RNA (1 mg/mL) was reverse transcribed using a SuperScript II kit for cDNA synthesis, Takara Bio Inc., Kyoto, Japan. The cDNA was subjected to quantitative real-time (qRT)-PCR using thermocyclers from Applied Biosystems (Franklin Lakes, NJ). The sequences of the primers to amplify the BPH-associated genes analyzed in this study are provided in Table 1.

**Table 1.** Quantitative real-time PCR primer sequences.

Target gene	Sequence	
5α-reductase type 2 (Rat)	Sense	5′-GACCACAGGCGAGATGCAGA-3′
	Antisense	5′-TGTGTTTCCCGTAACTGGCG-3′
FGF-2 (Rat)	Sense	5′-GAACCGGTACCTGGCTATGA-3′
	Antisense	5′-CCGTTTTGGATCCGAGTTTA-3′
PCNA (Rat)	Sense	5′-CAATTTCTAGCAACGCCTAAGAT-3′
	Antisense	5′-AAGAGGAAGCTGTGTCCATAGAG-3′
IL-1β (Rat)	Sense	5′-TCCTCTGTGACTCGTG GGAT-3′
	Antisense	5′-TCAGACAGCACGAGGCATTT-3′
IL-6 (Rat)	Sense	5′-AGAGACTTCCAGCCAGTTGC-3′
	Antisense	5′-AGCCTCCGACTTGTGAAGTG-3′
TNF-α (Rat)	Sense	5′-TCGTCTACTCCTCAGAGCCC-3′
	Antisense	5′-ACTTCAGCGTCTCGTGTGTT-3′
COX-2 (Rat)	Sense	5′-AATCGCTGTACAAGCAGTGG-3′
	Antisense	5′-GCAGCCATTTCTTTCTCTCC-3′
β-actin (Rat)	Sense	5′-CGTGAAAAGATGACCCAGAT-3′
	Antisense	5′-ACCCTCATAGATGGGCACA-3′
AR (Human)	Sense	5′-CTCACCAAGCTCCTGGACTC-3′
	Antisense	5′-CAGGCAGAAGACATCTGAAAG-3′
PSA (Human)	Sense	5′-GCAGCATTGAACCAGAGGAG-3′
	Antisense	5′-AGAACTGGGGAGGCTTGAGT-3′
β-actin (Human)	Sense	5′-GATGAGATTGGCATGGCTT-3′
	Antisense	5′-CACCTTCACCGTTCCAGTTT-3′

### Cell culture

Human prostate epithelial cell line, BPH-1, was purchased from DSMZ (Braunschweig, Germany). BPH-1 were maintained with Roswell Park Memorial Institute (RPMI) 1640 medium containing 10% FBS (Welgene, Daegu, Korea), 1% penicillin/streptomycin (Welgene) and insulin (10 mg/mL) at 37 °C, 5% CO_2_. Medium was replaced 2–3 times a week. The cells were subcultured using trypsin-EDTA (Welgene Daegu, Korea) to detach them from the culture dish and centrifuged at 800 rpm, 2 min. Subculture was progressed when the cells were 70 ∼ 80% full.

### MTT assay

MTT assay was used to determine the cell viability. After BPH-1 cells had been seeded 5 × 10^3^ cells/well using a hemocytometer. Culture (100 μL) was put into each well of a 96-well plate. After incubating the cells for 24 h, the medium was replaced by fresh and dissolved LCW was treated. MTT was prepared as a stock solution (5 mg/mL) in distilled water, filtered, and the MTT solution was added to each well. After incubation for 4 h at 37 °C, 5% CO_2_, the solution was removed in each well and DMSO (100 μL) was added to each well. The 96-well plates were read by a multi reader at 550 nm for absorbance density values to determine the cell viability and the percentage of surviving cells was calculated from the ratio of absorbance of treated to untreated cells

### Statistical analyses

Representative data from three independent experiments are presented as mean ± standard error of the mean (SEM). The significance of differences between control and experimental values were evaluated by one-way ANOVA. Analyses were performed with Graphpad Prism 5 (GraphPad Software Inc. CA, USA). Statistical significance was defined as *p* < 0.05.

## Results

### Effect of LCW on body and prostate weight in TPH rats

We used testosterone to induce prostate hyperplasia in rats for 6 weeks and then measured the weights of the body and prostate. As shown in Table 2, body weight was not significantly different before and after testosterone treatment. Administration of testosterone remarkably increased prostate weight compared to the normal control group. Oral administration of LCW at 50 and 100 mg/kg significantly reduced prostate weight compared to the testosterone-treated group (TPH) (*p* < 0.05). Administration of finasteride (10 mg/kg), a positive control, also had an inhibitory effect on prostate weight compared to the TPH group. However, the effect of LCW was not dose-dependent. The inhibition was 8.3, 80, 41.7 and 84.6% for LCW 50, 100, 200 mg/kg, and finasteride (10 mg/kg), respectively, compared to the TPH group. Moreover, rats with TPH had a much higher prostatic index than the normal control group. Inhibition of the prostatic index was 11.47, 69.78, 22.51 and 75.10% by LCW 50, 100, 200 mg/kg, and finasteride (10 mg/kg), respectively, compared to the TPH group (As shown in Table 2).

**Table 2.** Body weight, prostate weight and prostatic index in TPH rats.

Group	Body weight (g)		Prostate weight (g/kg body weight)	% Inhibition	Prostatic index × 10^−3^	% Inhibition
	Initial	Final				
NC	363.4 ± 5.2	512.5 ± 8.6	1.1 ± 0.2	–	2.141 ± 0.709	–
TPH	355.5 ± 4.3	492.8 ± 10.9	1.6 ± 0.2^a^	–	3.24 ± 0.669^a^	–
LCW-25	352.7 ± 4.7	467 ± 5.1	1.5 ± 0.1	8.3	3.21 ± 0.358	11.47
LCW-50	354 ± 9	461.7 ± 9.3	0.8 ± 0.04^b^	80	1.732 ± 0.103^b^	69.78
LCW-100	330 ± 8.3	474 ± 26.5	1.1 ± 0.2^b^	41.7	2.427 ± 0.813^b^	22.51
Finasteride	336.3 ± 5.8	466.4 ± 24.5	0.6 ± 0.1^b^	84.6	1.307 ± 0.37^b^	75.10

Values are expressed as mean ± S.E.M. Data were analyzed by one-way ANOVA followed by Bonferroni′s multiple comparison tests.

a *p* < 0.05, significantly different from the normal control group.

b *p* < 0.05, significantly different from the TPH group.

### Effects of LCW on histological features of the prostate in TPH rats

**Figure 1.**
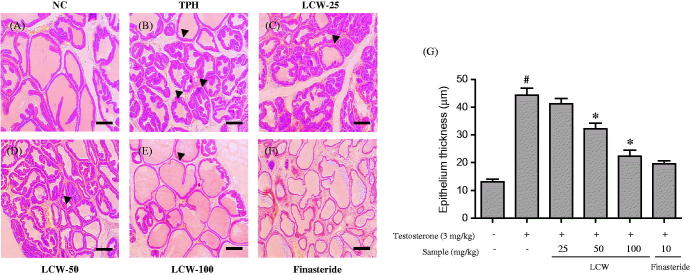
Effect of LCW on prostatic hyperplasia. (A) Normal control group, (B) TPH group, (C) LCW 25 mg/kg group, (D) LCW 50 mg/kg group, (E) LCW 100 mg/kg group, (F) finasteride 10 mg/kg group. (A–F) Prostate tissues were fixed, embedded in paraffin, and sectioned at 4 μm. Hematoxylin and eosin (H&E)-stained slides were visualized at × 100 magnification. (G) Area and length of prostatic epithelial thickness in H&E-stained slides were analyzed using Image J software (NIH, Maryland, USA), and average thicknesses were estimated by dividing areas by lengths. ^#^*p* < 0.05, significantly different from the normal control group. *p < 0.05, significantly different from the TPH group.

The inhibitory effects of LCW on the morphology of the prostate in testosterone-induced rats are demonstrated in Figure 1(A). Normal histological features of the prostate were observable in the normal control group, such as the presence of tubules of variable diameter with irregular lumens (Figure 1(A)). In the TPH group, the tubules were wider and the tubule walls were thickened. In addition, almost all tubules developed involutions in the lumens, inhibiting the volume of the lumen because of overgrowth and thickening of the epithelial cell layers, compared to the normal control group (Figure 1(B)). However, the changes in morphology of the prostate in the TPH group were ameliorated by oral administration of LCW or finasteride. Oral administration of LCW at 50 and 100 mg/kg restored the overall structure of the prostate compared to the TPH group and preserved the normal histological features of the prostate (Figure 1(D,E)). Finasteride also protected against changes in the overall structure of the prostate and preserved the normal histological features of the prostate (Figure 1(F)). The thickness of the epithelial layer was measured and found to be significantly attenuated by oral administration of LCW 50, 100 mg/kg or finasteride at 27, 49.5 and 55.5 μm, respectively (Figure 1(G)). These data indicated that LCW had an inhibitory effect on prostate hyperplasia in testosterone-induced rats.

### Effects of LCW on the serum level of DHT and transcript level of 5α-reductase in TPH rats

**Figure 2.**
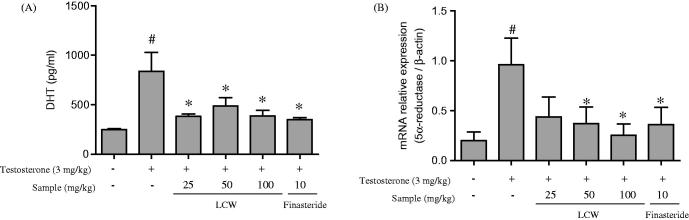
Effects of LCW on DHT and 5α-reductase expression in TPH rats. (A) DHT level was quantified by serum rat ELISA assay. (B) 5α-reductase expression was quantified by real-time PCR. Data are mean ± S.E.M. (*n* = 6). ^#^*p* < 0.05, significantly different from the normal control group. *p < 0.05, significantly different from the TPH group.

We investigated whether LCW is capable of regulating DHT production and 5α-reductase transcription in TPH rat model. Serum DHT level was significantly increased by testosterone. However, oral administration of LCW remarkably inhibited serum DHT level compared to the TPH group in a dose-dependent manner (Figure 2(A)). In addition, the increased expression of 5α-reductase induced by testosterone was significantly attenuated by oral administration of LCW at 50 and 100 mg/kg compared to the TPH group (Figure 2(B)). Treatment with finasteride as a positive control also had an inhibitory effect on testosterone-induced DHT production and 5α-reductase expression. These results confirmed that oral administration of LCW restored the normal morphology of the prostate in TPH by inhibiting the testosterone-induced increase in DHT serum level and 5α-reductase expression.

### Effect of LCW on the expression of inflammatory cytokines in the prostates of TPH rats

**Figure 3.**
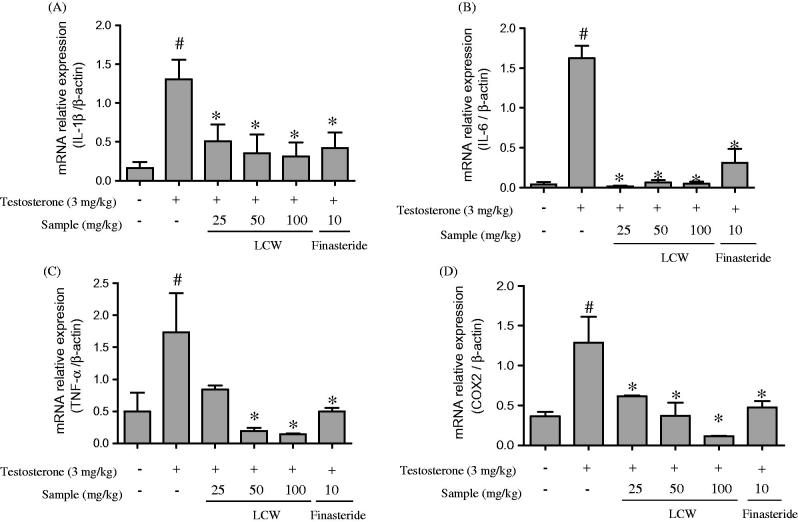
Effect of LCW on inflammatory cytokines in TPH. mRNA expression of IL-1β (A), IL-6 (B), TNF-α (C) and COX2 (D) in the prostate tissue of testosterone-treated rats administered LCW orally as quantified by quantitative real-time PCR. Values are mean ± S.E.M. (*n* = 6). ^#^*p* < 0.05, significantly different from the normal control group. *p < 0.05, significantly different from the TPH.

Previous studies have reported that chronic inflammation is associated with increased prostate volume and the pathogenesis and progression of BPH (Nickel et al. 2008). To investigate whether LCW could regulate the expression of inflammatory cytokines in the prostate of TPH rats, the gene expression of inflammatory cytokines was examined by qRT-PCR. TPH significantly elevated mRNA expression of IL-1β, IL-6, TNF-α and COX-2 compared to the normal control. Oral administration of LCW (50, 100 and 200 mg/kg) and finasteride 10 mg/kg remarkably ameliorated the testosterone-induced increase in mRNA expression of these inflammatory cytokines (Figure 3).

### Effects of LCW on the expression of PCNA and FGF-2 in the prostate of TPH rats

**Figure 4.**
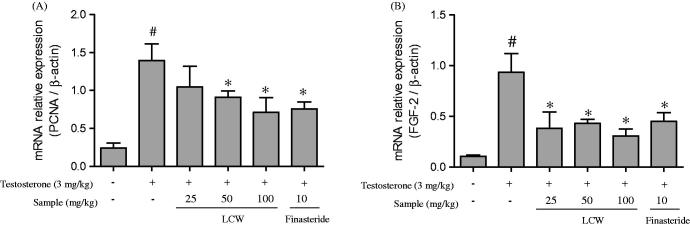
Effects of LCW on mRNA expression of PCNA and FGF-2 in TPH tissues. Transcript levels of (A) PCNA and (B) FGF-2 from prostate tissues of testosterone-treated rats with BPH that received oral administration of LCW as quantified by quantitative real-time PCR. Values are mean ± S.E.M. (*n* = 6). ^#^*p* < 0.05, significantly different from the normal control group. *p < 0.05, significantly different from the TPH group.

Increased prostatic cell growth is one of the main characteristics of BPH in elderly men. Proliferating cell nuclear antigen (PCNA) is a marker of BPH and is involved in progression of the G1/S phase of the cell cycle (Lai et al. 2004). In addition, fibroblast growth factors (FGFs) may play a role in prostate proliferation, as they have mitogenic and angiogenic activities (Xu et al. 2014). Orchiectomy and administration of testosterone significantly increased mRNA expression of PCNA and FGF-2 in the TPH group compared to the normal control group. However, oral administration of LCW (25, 50, 100 mg/kg) and 10 mg/kg finasteride remarkably attenuated the testosterone-induced increase in PCNA mRNA expression by 24.94, 34.52, 48.97 and 45.56, respectively, compared to the TPH group (Figure 4(A)). Furthermore, mRNA expression of FGF-2 was dose-dependently inhibited by oral administration of LCW and finasteride at about 59.08, 53.98, 67.14 and 51.77, respectively, compared to the TPH group (Figure 4(B)).

### Effect of LCW on mRNA expression of the androgen receptor (AR) and prostate-specific antigen (PSA) in BPH-1 cells

**Figure 5.**
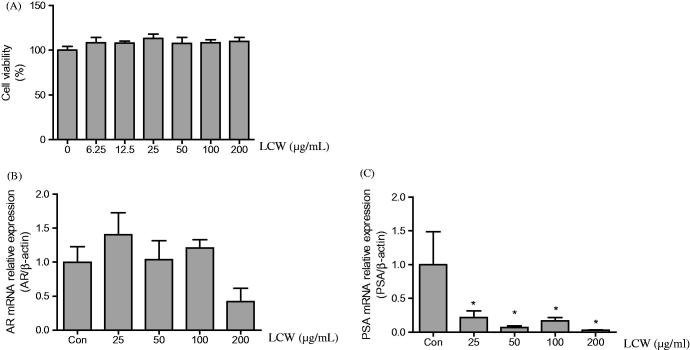
Effect of LCW on the mRNA expression of AR and PSA in BPH-1 cells. BPH-1 cells were treated with different concentrations of LCW for 24 h. (A) Cell viability was measured by MTT assay. mRNA expression of AR (B) and PSA (C) was quantified by quantitative real-time PCR. Data are mean ± S.E.M from three independent experiments. *p < 0.05, significantly different from the untreated group.

To confirm that treatment of LCW had an inhibitory effect on BPH, we used the benign prostatic hyperplasia epithelial cell line (BPH-1). BPH-1 cells were treated with LCW at concentrations of 6.25, 12.5, 25, 50, 100 and 200 μg/mL for 24 h. Then, the viability of BPH-1 cells was examined using the MTT assay. As shown in Figure 5(A), treatment with LCW did not affect the viability of BPH-1 cells.

AR signaling plays a critical role in enhancing cell growth in prostatic epithelial cells, thus promoting the development of BPH (Izumi et al. 2013). PSA is known to be one of the main downstream target genes of the AR and is used as a biomarker to investigate the progression of prostate cancer and BPH. Therefore, we investigated whether LCW treatment downregulated the expression of AR and PSA in BPH-1 cells by qRT-PCR. Treatment of cells with the highest concentration of LCW for 24 h significantly decreased the mRNA expression of AR to about 63% of the control (Figure 5(B)). In addition, mRNA expression of PSA was inhibited in a concentration-dependent manner by LCW compared to the control (Figure 5(C)).

### HPLC analysis

**Figure 6.**
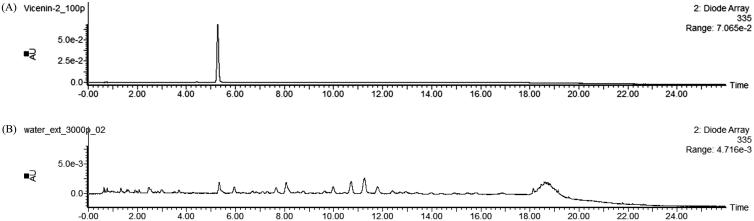
HPLC chromatogram for standard Vicenin-2 (A) and LCW (B). Both peaks have similar retention time of about 5.32 indicating that our LCW contains Vicenin-2.

The confirmation of Vicenin-2 was carried out using UV/Vis spectroscopic measurement by comparing the HPLC chromatogram of LCW with that of standard Vicenin-2. Specificity was established by lack on interfering peaks at the retention time for 5.32 min for both sample and the standard (Figure 6(A,B)). The calibration curve for standard was found to be linear over regression coeffcient (*R*^2^) of 0.99998. The content of Vicenin-2 in the LCW was analyzed 0.98 mg/g.

## Discussion

In this research, we investigated the effect of *L. cuneata* aqueous extract on testosterone-induced BPH in rats. We found that LCW attenuated the testosterone-induced increase in prostate weight and changes in histology of prostate tissue by decreasing expression of DHT and 5α-reductase, as well as the expression of markers of inflammation and oxidative stress in our rat model. In addition, expression of AR and PSA was inhibited at the transcriptional level by treatment of BPH-1 cells with LCW. The genotoxic experiment was commissioned to Korean Conformity Labratories (GT17-00036, GT17-00037 and GT17-00038). Since the LCW was reacted in male reproductive organs like prostate, it was necessary to check genetic toxicity to those organs. The results of genetic toxicity showed that LCW have no genotoxic (Supplement 1–6).

BPH is the most common male benign proliferative disorder. It is found in approximately 40% of 70-year-old men, with microscopic foci found in up to 80% of 70-year-old men. Excess secretion of androgens is the main factor underlying the development of BPH (Ekman 1989; Izumi et al. 2013). In the current study, testosterone significantly increased prostate weight and the prostatic index, and histological changes in prostate tissue consistent with BPH were observed in testosterone-treated rats compared to the normal control group. These observations indicated that our animal model was suitable for evaluating the effects of LCW on TPH. Oral administration of LCW significantly inhibited prostate weight and the prostatic index and had similar effects to finasteride, a drug currently used to treat TPH. Histological examination of the prostate tissue of testosterone-induced rats revealed thick epithelial layers, stromal proliferation and glandular hyperplasia. However, TPH rats that received LCW showed a marked decrease in epithelial layer thickness and mild glandular hyperplasia, suggesting that LCW is an effective treatment for TPH.

Testosterone and DHT play an important role in the development of male reproductive organs and are involved in the pathogenesis of BPH (Andriole et al. 2004; Miller & Tarter 2009). Serum concentrations of testosterone and DHT may change with age (Izumi et al. 2013). Serum DHT levels in BPH patients are remarkably higher than those in unaffected men of a similar age (Horton et al. 1975). DHT is mainly synthesized in the prostate, hair follicles and testes from circulating testosterone by the enzymatic action of 5α-reductase. DHT has greater binding affinity for ARs than testosterone and adrenal androgens (Andriole et al. 2004). Thus, a number of studies have investigated how DHT level is regulated by 5α-reductase. Finasteride is a representative 5α-reductase inhibitor used to treat BPH and downregulates testosterone and DHT levels in serum and the prostate gland, resulting in a decrease in prostate size and BPH-related LUTS (Bullock & Andriole Jr 2006; Paba et al. 2011). Researchers, however, are actively investigating alternative materials to finasteride to treat BPH because long-term use of finasteride has serious adverse effects (Cauci et al. 2017). In our experiments, the testosterone-induced increase in expression of serum DHT and 5α-reductase was significantly inhibited by oral administration of LCW in a dose-dependent manner. This suggests that the inhibitory effect of LCW in our TPH animal model was due to downregulation of serum DHT level and transcript level of 5α-reductase, suggesting that LCW may be a viable alternative to finasteride to treat TPH.

Inflammation is emerging as a critical factor in the etiopathogenesis of BPH (Kramer et al. 2007). The majority of BPH tissues have a vastly increased T leukocyte population, frequently express markers of activation, and show clustering of BPH-infiltrating T cells, suggesting a progressing immune response in BPH (Theyer et al. 1992). In addition, leukocytes that have infiltrated into prostate tissue release a variety of inflammatory cytokines, such as IL-1β, IL-2, IL-4, IL-6, IFN-γ and TNF-α, which play an important role in maturation of the stroma and development of stromal nodules in BPH (Kramer et al. 2002). Many research groups have been investigating compounds that inhibit the release of inflammatory cytokines as potentially effective therapeutic agents to treat BPH. In the present study, expression of the inflammatory cytokines IL-1β, IL-4, TNF-α and COX-2, an enzyme responsible for inflammation and pain, was significantly increased in the prostate tissue of rats treated with testosterone. However, treatment with LCW suppressed mRNA expression of IL-1β, IL-4, TNF-α and COX-2 in a dose-dependent manner compared to the testosterone-treated group. Extracts and bio-compounds of *L. cuneata* have already been shown to have anti-inflammatory effects (Lee et al. 2013, 2016). In the current study, we established that LCW had an anti-inflammatory effect in a TPH rat model, suggesting that it may be an effective therapeutic agent for TPH.

Evidence from cell culture systems as well as immunohistochemical and mRNA analyses of BPH tissue, such as expression profiling of growth-regulatory proteins and cytokines, has provided insights into the potential roles of these growth factors and cytokines in the pathogenesis of BPH. In addition, several growth factors and a variety of interleukins that interact may cause abnormal stromal and epithelial cell growth in the prostate (Lucia & Lambert 2008). Two types of FGFs found in BPH tissue are FGF-2 and basic FGF (bFGF). FGF-2 is 2- to 3-fold upregulated in BPH tissue compared to normal tissue (Ropiquet et al. 1999). Expression of the principal receptor for FGF-2, FGFR1, is also elevated in BPH, suggesting upregulation of the FGF-2 signaling pathway in BPH (Boget et al. 2001). Finasteride’s mode of action is based on the involvement of FGF-2 in BPH. BPH treated with finasteride was associated with a reduction in FGF-2 level in comparison to untreated BPH, indicating that regulation of FGF-2 in BPH is dependent, at least in part, on DHT (Sáez et al. 1999). In the present study, the testosterone-induced increase in mRNA expression of FGF-2 in the prostate tissue of rats was significantly inhibited by oral administration of LCW in a dose-dependent manner, indicating that LCW has the potential to treat TPH.

PCNA is widely used to evaluate cellular proliferation in benign and malignant proliferating tissues, and PCNA level has been revealed to correlate directly with the proliferate state of various tissues, including the prostate (Zhong et al. 2008; Vikram et al. 2011). Consistent with previous studies, testosterone-induced BPH in rats was characterized by a significant increase in mRNA expression of PCNA. However, oral administration of LCW markedly inhibited the mRNA expression of PCNA compared to the TPH group, suggesting suppression of prostatic hyperplasia.

In the present study, we used the BPH-1 cell line, a cell type established from human benign prostate hyperplasia, to confirm the effects of LCW *in vitro*. BPH-1 cells overexpress the AR and PSA, a pivotal downstream target gene of the AR. The overexpression of AR and PSA in BPH-1 cells was significantly reduced by treatment with LCW in a concentration-dependent manner compared to the untreated group. Although the doses used in the *in vitro* experiments did not inhibit testosterone-induced cell growth, AR and PSA were effectively inhibited. *In vitro*, at very high doses, there was nonspecific cytotoxicity with testosterone, and *in vivo* tests were performed at doses expected to have no other side effects. Also, we confirmed that the content of vicenin-2 is 0.89 mg/g in LCW. Vicenin-2 has been reported to have anti-inflammatory effects (Kang et al. 2015) and prostatic cancer prevention (Nagaprashantha et al. 2011; Sharad et al. 2017). In the future, it is necessary to identify substances showing nonspecific cytotoxicity and substances showing specific effects on testosterone receptors. Finally, our results indicate that LCW had a protective effect against BPH both *in vitro* and *in vivo*. However, further research is required to identify the precise molecular mechanism by which LCW reduces the inflammation associated with prostatic hyperplasia and to determine the protective efficacy of discrete components of LCW.

## Conclusions

Our results provide the first demonstration that the aqueous extract of *L. cuneata* may be effective at treating prostatic hyperplasia. LCW successfully inhibited the symptoms of TPH in rats by decreasing the expression of DHT, 5α-reductase, and inflammation. The PCNA gene, which induces abnormal cell growth, was inhibited by LCW administration, and the growth factor FGF-2 was also inhibited. Therefore, it was concluded that administration of LCW inhibited the enlargement of the prostate tissue due to hormone changes induced by testosterone induction. In addition, treatment with LCW significantly attenuated transcription of the AR and PSA in BPH-1 cells. These findings strongly suggest that LCW is a novel candidate for the treatment of BPH as a functional food supplement. However, further studies are necessary to analyze active compounds and identify the molecular mechanisms underlying the treatment efficacy of LCW in prostatic hyperplasia.

## Supplementary Material

Corrected_supplement_data.docx

## References

[CIT0001] Ahn D 1998 Illustrated book of Korean medicinal herbs. Seoul: Kyohaksa.

[CIT0002] Andriole G, Bruchovsky N, Chung LW, Matsumoto AM, Rittmaster R, Roehrborn C, Russell D, Tindall D 2004 Dihydrotestosterone and the prostate: the scientific rationale for 5alpha-reductase inhibitors in the treatment of benign prostatic hyperplasia. J Urol. 172:1399–1403. 15371854 10.1097/01.ju.0000139539.94828.29

[CIT0003] Babu SV, Veeresh B, Patil AA, Warke Y 2010 Lauric acid and myristic acid prevent testosterone induced prostatic hyperplasia in rats. Euro Pharm. 626:262–265. 10.1016/j.ejphar.2009.09.037 19786012

[CIT0004] Boget S, Cereser C, Parvaz P, Leriche A, Revol A 2001 Fibroblast growth factor receptor 1 (FGFR1) is over-expressed in benign prostatic hyperplasia whereas FGFR2-iiic and FGFR3 are not. Euro Endocrinol. 145:303–310. 10.1530/eje.0.1450303 11517011

[CIT0005] Bullock TL, Andriole GL Jr. 2006 Emerging drug therapies for benign prostatic hyperplasia. Expert Opin Emerg Drugs. 11:111–123. 16503830 10.1517/14728214.11.1.111

[CIT0006] Cauci S, Chiriaco G, Cecchin E, Toffoli G, Xodo S, Stinco G, Trombetta C 2017 Androgen receptor (AR) gene (CAG)n (GGN)n length polymorphisms and symptoms in young males with long lasting adverse effects after finasteride use against androgenic lopecia. Sex Med. 5:e61–e71. 28024997 10.1016/j.esxm.2016.11.001 PMC5302381

[CIT0007] Deng F, Chang J, Zhang JS 2007 New flavonoids and other constituents from *Lespedeza cuneata*. J Asian Nat Prod Res. 9:655–658. 17943561 10.1080/10286020600979894

[CIT0008] Ding JL, Lim IJ, Lee HD, Cha WS 2006 Analysis of minerals, amino acids, and vitamin of *Lespedeza cuneata*. KSBB J. 21:414–417.

[CIT0009] Ekman P 1989 BPH epidemiology and risk factors. Prostate Suppl. 2:23–31. 2482771 10.1002/pros.2990150505

[CIT0010] Gacci M, Corona G, Salvi M, Vignozzi L, McVary KT, Kaplan SA, Roehrborn CG, Serni S, Mirone V, Carini M, et al. 2012 A systematic review and meta-analysis on the use of phosphodiesterase 5 inhibitors alone or in combination with α-blockers for lower urinary tract symptoms due to benign prostatic hyperplasia. Eur Urol. 61:994–1003. 22405510 10.1016/j.eururo.2012.02.033

[CIT0011] Gilbert TD Jr, Davis EA, Ollendorf DA 2006 An examination of treatment patterns and costs of care among patients with benign prostatic hyperplasia. Am J Manag Care. 12:S99–S110. 16551208

[CIT0012] Gravas S, Oelke M 2010 Current status of 5alpha-reductase inhibitors in the management of lower urinary tract symptoms and BPH. World J Urol. 28:9–15. 19956956 10.1007/s00345-009-0493-y PMC2809314

[CIT0013] Horton R, Hsieh P, Barberia J, Pages L, Cosgrove M 1975 Altered blood androgens in elderly men with prostate hyperplasia. J Clin Endocrinol Metab. 41:793–796. 1176586 10.1210/jcem-41-4-793

[CIT0014] Huang KC 1998 The pharmacology of Chinese herbs. Taylor&Francis Group.

[CIT0015] Izumi K, Mizokami A, Lin WJ, Lai KP, Chang C 2013 Androgen receptor roles in the development of benign prostate hyperplasia. Am J Pathol. 182:1942–1949. 23570837 10.1016/j.ajpath.2013.02.028 PMC3668026

[CIT1015] Kang H, Ku SK, Jung B, Bae JS 2015 Anti-inflammatory effects of vicenin-2 and scolymoside *in vitro* and *in vivo*. Inflamm Res. 64:1005–1021. 26482935 10.1007/s00011-015-0886-x

[CIT0016] Kim JS, Kim MJ 2010 *In vitro* anti-oxidant activity of *Lespedeza cuneata* methanolic extracts. J Med Plants Res. 4:674–679.

[CIT0017] Kramer G, Mitteregger D, Marberger M 2007 Is benign prostatic hyperplasia (BPH) an immune inflammatory disease? Eur Urol. 51:1202–1216. 17182170 10.1016/j.eururo.2006.12.011

[CIT0018] Kramer G, Steiner GE, Handisurya A, Stix U, Haitel A, Knerer B, Gessl A, Lee C, Marberger M 2002 Increased expression of lymphocyte-derived cytokines in benign hyperplastic prostate tissue, identification of the producing cell types, and effect of differentially expressed cytokines on stromal cell proliferation. Prostate. 52:43–58. 11992619 10.1002/pros.10084

[CIT0019] Lai J, Xia Q, Zhang X, Zhao G, Xu S, Zheng D 2004 [Expression and significance of PCNA and p27 in benign prostate hypertrophy and prostate carcinoma]. Zhonghua Zhong Liu Za Zhi. 26:476–478. Chinese. 15555337

[CIT0020] Lee H, Jung JY, Hwangbo M, Ku SK, Kim YW, Jee SY 2013 Anti-inflammatory effects of *Lespedeza cuneata in vivo* and *in vitro*. Korea J Herbol. 28:83–92.

[CIT0021] Lee SJ, Hossaine MA, Park SC 2016 A potential anti-inflammation activity and depigmentation effect of *Lespedeza bicolor* extract and its fractions. Saudi Biol Sci. 23:9–14. 10.1016/j.sjbs.2015.01.016 PMC4705262 26858533

[CIT0022] Lucia MS, Lambert JR 2008 Growth factors in benign prostatic hyperplasia: Basic science implications. Curr Urol Rep. 9:272–278. 18765125 10.1007/s11934-008-0048-6

[CIT0023] Matsuura S, Iinuma M, Ito E, Takami H, Kage K 1978 [Studies on the constituents of the useful plants. VIII. The constituents of *Lespedeza cuneata* G. Don (author's transl)]. Yakugaku Zasshi. 98:1542–1544. Japanese. 739395 10.1248/yakushi1947.98.11_1542

[CIT0024] McVary KT 2007 A review of combination therapy in patients with benign prostatic hyperplasia. Clin Ther. 29:387–398. 17577460 10.1016/s0149-2918(07)80077-4

[CIT0025] Miller J, Tarter T 2009 Combination therapy with dutasteride and tamsulosin for the treatment of symptomatic enlarged prostate. Clin Inter Aging. 4:251–258. 10.2147/cia.s4102 PMC2697590 19554096

[CIT1026] Nagaprashantha LD, Vatsyayan R, Singhal J, Fast S, Roby R, Awasthi S, Singhal SS 2011 Anti-cancer effects of noverl flavonoid vicenin-2 as a single agent and in synergistin combination with docetaxel in prostate cancer. Biochem Pharmacol. 82:1100–1109. 21803027 10.1016/j.bcp.2011.07.078 PMC3252753

[CIT0026] Nickel JC, Roehrborn CG, O'Leary MP, Bostwick DG, Somerville MC, Rittmaster RS 2008 The relationship between prostate inflammation and lower urinary tract symptoms: examination of baseline data from the reduce trial. Eur Urol. 54:1379–1384. 18036719 10.1016/j.eururo.2007.11.026 PMC2643127

[CIT0027] Paba S, Frau R, C Godar S, Devoto P, Marrosu F, Bortolato M 2011 Steroid 5α-reductase as a novel therapeutic target for schizophrenia and other neuropsychiatric disorders. Curr Pharm Design. 17:151–167. 10.2174/138161211795049589 21361868

[CIT0028] Ropiquet F, Giri D, Lamb DJ, Ittmann M 1999 FGF7 and FGF2 are increased in benign prostatic hyperplasia and are associated with increased proliferation. J Urol. 162:595–599. 10411093

[CIT0029] Rudkowska I, AbuMweis SS, Nicolle C, Jones PJ 2008 Cholesterol-lowering efficacy of plant sterols in low-fat yogurt consumed as a snack or with a meal. J Am Coll Nutr. 27:588–595. 18845709 10.1080/07315724.2008.10719742

[CIT0030] Sáez C, González Baena AC, Japón MA, Giráldez J, Segura DI, Rodríguez Vallejo JM, González Esteban J, Miranda G, Torrubia F 1999 Expression of basic fibroblast growth factor and its receptors FGFR1 and FGFR2 in human benign prostatic hyperplasia treated with finasteride. Prostate. 40:83–88. 10386468 10.1002/(sici)1097-0045(19990701)40:2<83::aid-pros3>3.0.co;2-n

[CIT4031] Sharad SS, Divya J, Preeti S, Sanjay A, Jyotsana S, David H 2017 Targeting the mercapturic acid pathway and vicenin-2 for prevention of prostatic cancer. Biochim Biophys Acta Rev Cancer. 1868:167–175. 28359741 10.1016/j.bbcan.2017.03.009 PMC5638116

[CIT0031] Shin IS, Lee MY, Ha HK, Seo CS, Shin HK 2012 Inhibitory effect of yukmijihwang-tang, a traditional herbal formula against testosterone-induced benign prostatic hyperplasia in rats. BMC Complement Alter Med. 12:48–54. 10.1186/1472-6882-12-48 PMC3457905 22520510

[CIT0032] Theyer G, Kramer G, Assmann I, Sherwood E, Preinfalk W, Marberger M, Zechner O, Steiner G 1992 Phenotypic characterization of infiltrating leukocytes in benign prostatic hyperplasia. Lab Invest. 66:96–107. 1370561

[CIT0033] Vikram A, Kushwaha S, Jena G 2011 Relative influence of testosterone and insulin in the regulation of prostatic cell proliferation and growth. Steroids. 76:416–423. 21215763 10.1016/j.steroids.2010.12.014

[CIT0034] Wilt TJ, Ishani A, MacDonald R, Stark G, Mulrow CD, Lau J 2000 Beta sitosterols for benign prostatic hyperplasia. Cochrane Database Syst Rev. (2):CD001043. 10.1002/14651858.CD001043 PMC8407049 10796740

[CIT0035] Xu DH, Wang LH, Mei XT, Li BJ, Lv JL, Xu SB 2014 Protective effects of seahorse extracts in a rat castration and testosterone-induced benign prostatic hyperplasia model and mouse oligospermatism model. Environ Toxicol Pharm. 37:679–688. 10.1016/j.etap.2014.02.001 24607683

[CIT0036] Zhong W, Peng J, He H, Wu D, Han Z, Bi X, Dai Q 2008 Ki-67 and PCNA expression in prostate cancer and benign prostatic hyperplasia. Clin Invest Med. 31:8–15. 10.25011/cim.v31i1.3136 18312749

