# Supplementary material for: Effects of Lespedeza Cuneata aqueous extract on testosterone-induced prostatic hyperplasia
Source: Pharm Biol. 2019 Feb 6;57(1):90–8. doi: 10.1080/13880209.2018.1564929 (PMC6366415; doi:10.1080/13880209.2018.1564929)
Supplement: Corrected_supplement_data.docx [file IPHB_A_1564929_SM3696.docx]

**Supplement 1.** Microorganism reverting mutation test

| Metabolic activation | | Dose  (μg/plate) | Number of colony/plate | | | | |
| --- | --- | --- | --- | --- | --- | --- | --- |
|  |  |  | Base-pair substitution type | | | Frameshift type | |
|  |  |  | TA100 | TA1535 | WP2uvrA | TA98 | TA1537 |
| S9Mix(-) | | 0 | 124  130  132 | 6  6  6 | 35  38  43 | 38  39  39 | 5  4  5 |
|  |  | Mean±SD | 129±29n | 6±29n | 39±99n | 39±99n | 5±0.6 |
|  |  | 62 | 120  128  120 | 7  9  5 | 42  33  39 | 36  27  32 | 5  7  9 |
|  |  | Mean±SD | 123±23n | 7±23n | 38±83n | 32±23n | 5±1.0 |
|  |  | 185 | 122  116  120 | 10  8  6 | 35  39  36 | 36  27  32 | 5  7  9 |
|  |  | Mean±SD | 119±3.1 | 8±2.0 | 37±2.1 | 32±4.5 | 7±2.0 |
|  |  | 556 | 109  103  110 | 9  6  6 | 41  35  40 | 37  36  29 | 7  8  7 |
|  |  | Mean±SD | 107±3.8 | 7±1.7 | 39±3.2 | 34±4.4 | 7±0.6 |
|  |  | 1667 | 124  124  120 | 6  7  8 | 37  42  34 | 34  36  37 | 8  6  6 |
|  |  | Mean±SD | 123±2.3 | 7±1.0 | 38±4.0 | 36±1.5 | 7±1.2 |
|  |  | 5000 | 120  106  106 | 10  7  7 | 39  38  32 | 40  36  38 | 7  6  6 |
|  |  | Mean±SD | 111±8.1 | 8±1.7 | 36±3.8 | 38±2.0 | 6±0.6 |
| S9Mix(+) | | 0 | 112  122  114 | 13  19  13 | 49  52  36 | 38  36  32 | 17  16  13 |
|  |  | Mean±SD | 116±5.3 | 15±3.5 | 46±8.5 | 35±3.1 | 15±2.1 |
|  |  | 62 | 130  126  134 | 13  13  17 | 43  53  55 | 34  32  42 | 12  15  16 |
|  |  | Mean±SD | 130±4.0 | 14±2.3 | 50±6.4 | 36±5.3 | 14±2.1 |
|  |  | 185 | 124  112  118 | 17  13  14 | 55  33  47 | 35  28  28 | 18  13  18 |
|  |  | Mean±SD | 118±6.0 | 15±2.1 | 45±11.1 | 30±4.0 | 16±2.9 |
|  |  | 556 | 126  136  130 | 15  12  13 | 39  50  43 | 33  42  37 | 13  15  11 |
|  |  | Mean±SD | 131±5.0 | 13±1.5 | 44±5.6 | 37±4.5 | 13±2.0 |
|  |  | 1667 | 121  119  118 | 13  10  10 | 40  50  34 | 38  33  32 | 13  15  11 |
|  |  | Mean±SD | 119±1.5 | 11±1.7 | 41±5.1 | 34±3.2 | 13±2.0 |
|  |  | 5000 | 124  128  124 | 14  11  12 | 50  59  61 | 30  38  35 | 16  12  15 |
|  |  | Mean±SD | 125±2.3 | 12±1.5 | 57±5.9 | 34±4.0 | 14±2.1 |
| Positive  controls | S9Mix(-) | Positive | AF-2 | NaN_3_ | AF-2 | AF-2 | 9-AA |
|  |  | Dose | 0.01 | 0.5 | 0.01 | 0.1 | 80 |
|  |  | Number of colony | 472  456  470 | 247  293  304 | 351  367  338 | 438  363  387 | 1972  2036  2029 |
|  |  | Mean±SD | 466±8.7 | 281±30.2 | 352±14.5 | 396±38.3 | 2012±35.1 |
|  | S9Mix(+) | Positive | 2-AA | 2-AA | 2-AA | 2-AA | 2-AA |
|  |  | Dose | 1.0 | 2.0 | 10 | 0.5 | 2.0 |
|  |  | Number of colony | 348  328  303 | 294  266  310 | 411  494  488 | 360  492  454 | 298  256  286 |
|  |  | Mean±SD | 326±22.5 | 290±22.3 | 464±46.3 | 435±68.0 | 280±21.6 |

TA: *Samonella typhimurium*, WP2uvrA: Escherichia coli, S9: Aroclor 1254, AF-2: 2-(2-Furyl)-3-(5-nitro-2-furyl) acrylamide, NaN_3_: Sodium azide, 9-AA: 9-Aminoacridine hydrochloride hydrate, 2-AA: 2-Aminoanthracene.

**Supplement 2.** The number of cells with chromosome aberrations in the absence or presence of S9 mix

| Exposure^a)^ | S9 mix | Dose  (μg/ml) | Cell No. | Aberration | | | | | | No. of total chromosome aberrations | | No. of cells with chromosome aberrations | |
| --- | --- | --- | --- | --- | --- | --- | --- | --- | --- | --- | --- | --- | --- |
|  |  |  |  | Chromatid type | | Chromosome type | | PP/ER | Gap |  |  |  |  |
|  |  |  |  | ctb | cte | csb | cse |  |  | (-)Gap | (+)Gap | (-)Gap | (+)Gap |
| 24-0 | - | Negative control | 150 | 0 | 0 | 0 | 0 | 0 | 0 | 0 | 0 | 0 | 0 |
|  | - |  | 150 | 1 | 0 | 0 | 0 | 0 | 0 | 1 | 1 | 1 | 1 |
|  | - | 185.19 | 150 | 1 | 0 | 0 | 0 | 0 | 1 | 1 | 2 | 1 | 2 |
|  | - |  | 150 | 0 | 0 | 0 | 0 | 0 | 1 | 0 | 1 | 0 | 1 |
|  | - | 555.56 | 150 | 2 | 0 | 0 | 0 | 0 | 0 | 2 | 2 | 2 | 2 |
|  | - |  | 150 | 0 | 0 | 0 | 0 | 0 | 0 | 0 | 0 | 0 | 0 |
|  | - | 1666.67 | 150 | 0 | 0 | 0 | 0 | 0 | 0 | 0 | 0 | 0 | 0 |
|  | - |  | 150 | 0 | 1 | 0 | 0 | 0 | 0 | 1 | 1 | 1 | 1 |
|  | - | MMC | 150 | 6 | 30 | 0 | 0 | 0 | 0 | 36 | 36 | 36 | 36 |
|  | - |  | 150 | 3 | 35 | 0 | 0 | 0 | 0 | 38 | 38 | 38 | 38 |
| 6-18 | - | Negative control | 150 | 0 | 0 | 0 | 0 | 0 | 0 | 0 | 0 | 0 | 0 |
|  | - |  | 150 | 0 | 1 | 0 | 0 | 0 | 0 | 1 | 1 | 1 | 1 |
|  | - | 555.56 | 150 | 0 | 1 | 0 | 0 | 0 | 0 | 1 | 1 | 1 | 1 |
|  | - |  | 150 | 0 | 1 | 0 | 0 | 0 | 1 | 1 | 2 | 1 | 1 |
|  | - | 1666.67 | 150 | 0 | 0 | 0 | 0 | 0 | 0 | 0 | 0 | 0 | 0 |
|  | - |  | 150 | 1 | 0 | 0 | 0 | 0 | 1 | 1 | 2 | 1 | 2 |
|  | - | 5000 | 150 | 1 | 1 | 0 | 0 | 0 | 0 | 2 | 2 | 2 | 2 |
|  | - |  | 150 | 0 | 1 | 0 | 0 | 0 | 0 | 1 | 1 | 1 | 1 |
|  | - | MMC | 150 | 5 | 28 | 0 | 0 | 0 | 0 | 33 | 33 | 33 | 33 |
|  | - |  | 150 | 2 | 30 | 0 | 0 | 0 | 0 | 32 | 32 | 32 | 32 |
| 6-18 | + | Negative control | 150 | 1 | 0 | 0 | 0 | 0 | 0 | 1 | 1 | 1 | 1 |
|  | + |  | 150 | 0 | 0 | 0 | 0 | 0 | 0 | 0 | 0 | 0 | 0 |
|  | + | 555.56 | 150 | 0 | 1 | 0 | 0 | 0 | 0 | 1 | 1 | 1 | 1 |
|  | + |  | 150 | 1 | 0 | 0 | 0 | 0 | 0 | 1 | 1 | 1 | 1 |
|  | + | 1666.67 | 150 | 1 | 0 | 0 | 0 | 0 | 0 | 1 | 1 | 1 | 1 |
|  | + |  | 150 | 0 | 1 | 0 | 0 | 0 | 0 | 1 | 1 | 1 | 1 |
|  | + | 5000 | 150 | 0 | 1 | 0 | 0 | 0 | 0 | 1 | 1 | 1 | 1 |
|  | + |  | 150 | 1 | 0 | 0 | 0 | 0 | 0 | 1 | 1 | 1 | 1 |
|  | + | B[a]P | 150 | 5 | 33 | 0 | 0 | 0 | 0 | 38 | 38 | 38 | 38 |
|  | + |  | 150 | 4 | 33 | 0 | 0 | 0 | 0 | 37 | 37 | 37 | 37 |

B[a]P : Benzo[a]pyrene (20 μg/ml), ctb: Chromatid break, cte: Chromatid exchange, csb: Chromosome break, cse: Chromosomal crossover, PP: Polyploidy, ER: Endoreduplication, Gap: S9: Aroclor 1254

a) Treatment time-recovery time

**Supplement 3.** Test result of relative increase in cell counts (RICC) (preliminary range-finding test).

| Exposure^a)^ | S9 mix | Dose  (μg/ml) | Cell counts(ｘ10^6^) | | | | | RICC(%)^b)^ |
| --- | --- | --- | --- | --- | --- | --- | --- | --- |
|  |  |  | Plate A | | Plate B | | Mean |  |
| 24-0 | - | NC | 1.90 | 1.84 | 1.92 | 1.86 | 1.88 | 100.00 |
|  | - | 2.29 | 1.83 | 1.77 | 1.76 | 1.80 | 1.79 | 95.11 |
|  | - | 6.86 | 1.76 | 1.68 | 1.77 | 1.76 | 1.74 | 92.53 |
|  | - | 20.58 | 1.64 | 1.58 | 1.63 | 1.61 | 1.62 | 85.60 |
|  | - | 61.73 | 1.47 | 1.36 | 1.47 | 1.42 | 1.43 | 75.54 |
|  | - | 185.19 | 1.26 | 1.27 | 1.30 | 1.31 | 1.29 | 67.66 |
|  | - | 555.56 | 1.08 | 1.13 | 1.10 | 1.09 | 1.10 | 57.61 |
|  | - | 1666.67 | 0.94 | 0.91 | 0.90 | 1.00 | 0.94 | 48.78 |
|  | - | 5000 | 0.56 | 0.54 | 0.43 | 0.50 | 0.51 | 25.41 |
| 6-18 | - | NC | 1.76 | 1.69 | 1.73 | 1.75 | 1.73 | 100.00 |
|  | - | 2.29 | 1.61 | 1.59 | 1.55 | 1.51 | 1.57 | 90.10 |
|  | - | 6.86 | 1.56 | 1.54 | 1.46 | 1.55 | 1.53 | 87.89 |
|  | - | 20.58 | 1.42 | 1.51 | 1.55 | 1.54 | 1.51 | 86.56 |
|  | - | 61.73 | 1.41 | 1.45 | 1.38 | 1.44 | 1.42 | 81.54 |
|  | - | 185.19 | 1.37 | 1.34 | 1.31 | 1.32 | 1.34 | 76.51 |
|  | - | 555.56 | 1.29 | 1.20 | 1.23 | 1.29 | 1.25 | 71.64 |
|  | - | 1666.67 | 1.23 | 1.12 | 1.20 | 1.27 | 1.23 | 70.16 |
|  | - | 5000 | 0.94 | 0.97 | 0.82 | 0.84 | 0.89 | 50.37 |
| 6-18 | + | NC | 1.38 | 1.42 | 1.39 | 1.36 | 1.39 | 100.00 |
|  | + | 2.29 | 1.33 | 1.29 | 1.31 | 1.26 | 1.30 | 93.32 |
|  | + | 6.86 | 1.35 | 1.28 | 1.22 | 1.28 | 1.28 | 92.21 |
|  | + | 20.58 | 1.26 | 1.29 | 1.23 | 1.20 | 1.25 | 89.42 |
|  | + | 61.73 | 1.18 | 1.19 | 1.14 | 1.21 | 1.18 | 84.60 |
|  | + | 185.19 | 1.13 | 1.06 | 1.18 | 1.07 | 1.11 | 79.41 |
|  | + | 555.56 | 1.04 | 1.09 | 1.09 | 1.14 | 1.09 | 77.92 |
|  | + | 1666.67 | 1.03 | 1.05 | 0.90 | 1.01 | 1.00 | 71.06 |
|  | + | 5000 | 0.95 | 0.90 | 0.98 | 0.99 | 0.96 | 67.90 |

NC: Negative control, S9: Aroclor 1254

a) Treatment time-recovery time

b) RICC (Relative increase in cell counts, %) = Increase in number of cells in treated cultures (final-starting)/ Increase in number of cells in control cultures (final-starting) * 100

**Supplement 4.** Micronucleus test in animal

| Groups | | Dose (mg/kg, bw/day) | Animal No. | MNPCE/4000 PCEs (Mean ±SD, %) | PCE/(PCE+NCE)  (Mean±SD) |
| --- | --- | --- | --- | --- | --- |
| Negative control | Vehicle | 0 | 1 | 0.25 | 0.51 |
|  |  |  | 2 | 0.18 | 0.50 |
|  |  |  | 3 | 0.28 | 0.50 |
|  |  |  | 4 | 0.25 | 0.51 |
|  |  |  | 5 | 0.13 (0.22 ± 0.06) | 0.50 (0.50 ±0.01) |
| Test substance | LCW | 500 | 6 | 0.38 | 0.52 |
|  |  |  | 7 | 0.15 | 0.51 |
|  |  |  | 8 | 0.10 | 0.49 |
|  |  |  | 9 | 0.38 | 0.51 |
|  |  |  | 10 | 0.13 (0.23 ± 0.14) | 0.48 (0.50 ±0.02) |
|  |  | 1000 | 11 | 0.18 | 0.47 |
|  |  |  | 12 | 0.30 | 0.50 |
|  |  |  | 13 | 0.23 | 0.51 |
|  |  |  | 14 | 0.33 | 0.52 |
|  |  |  | 15 | 0.18 (0.24 ± 0.07) | 0.53 (0.50 ± 0.02) |
|  |  | 2000 | 16 | 0.23 | 0.48 |
|  |  |  | 17 | 0.23 | 0.49 |
|  |  |  | 18 | 0.23 | 0.50 |
|  |  |  | 19 | 0.23 | 0.50 |
|  |  |  | 20 | 0.33 (0.25 ± 0.04) | 0.50 (0.49 ± 0.02) |
| Positive control | MMC | 2.0 | 21 | 13.18 | 0.44 |
|  |  |  | 22 | 11.05 | 0.45 |
|  |  |  | 23 | 12.75 | 0.42 |
|  |  |  | 24 | 12.30 | 0.49 |
|  |  |  | 25 | 12.55 (12.37 ± 0.80)** | 0.50 (0.46 ± 0.04) |

Vehicle: Distilled water, MNPCE: Micronucleated polychromatic erythrocytes, PCE: Polychromatic erythrocyte, NCE: Normochromatic erythrocyte, MMC: Mitomycin C

** Significantly different from the control at *p*<0.01 (one-way ANOVA)

**Supplement 5.** Body weight of animals in Micronucleus test

| Groups | | Dose (mg/kg, bw/day) | Animal NO. | Body weights (gram, Mean ± S.D) | |
| --- | --- | --- | --- | --- | --- |
|  |  |  |  | Administration | Sacrifice |
| Negative control | Vehicle | 0 | 5 | 35.40 ± 1.40 | 36.75 ± 1.82 |
| Test substance | LCW | 500 | 5 | 35.12 ± 1.07 | 36.33 ± 1.60 |
|  |  | 1000 | 5 | 35.30 ± 1.13 | 36.90 ± 1.43 |
|  |  | 2000 | 5 | 35.34 ± 1.10 | 36.88 ± 1.03 |
| Positive control | MMC | 2.0 | 5 | 34.86 ± 1.05 | 35.98 ± 0.67 |

Vehicle: Distilled water

MMC: Mitomycin C

**Supplement 6.** Clinical signs in Micronucleus test

| Groups | | Dose (mg/kg, bw/day) | Routs | Animal NO. | Clinical signs | Days post dosing | | | | |
| --- | --- | --- | --- | --- | --- | --- | --- | --- | --- | --- |
|  |  |  |  |  |  | 1^st^ administration | | 2^nd^ administration | | 1 |
|  |  |  |  |  |  | Post dosnig | 3 hrs post dosing | Post dosing | 3 hr post dosing |  |
| Negative control | Vehicle | 0 | P.O | 5 | NAD | 5 | 5 | 5 | 5 | 5 |
| Test substance | LCW | 500 | P.O | 5 | NAD | 5 | 5 | 5 | 5 | 5 |
|  |  | 1000 | P.O | 5 | NAD | 5 | 5 | 5 | 5 | 5 |
|  |  | 2000 | P.O | 5 | NAD | 5 | 5 | 5 | 5 | 5 |
| Positive control | MMC | 2.0 | I.P | 5 | NAD | 5 | 5 | 5 | 5 | 5 |

P.O: per Os, I.P: Intraperitoneal, MMC: Mitomycin C, Vehicle: Distilled water, NAD: No Abnormalities Detected
